# A Coupled Phase-Temperature Model for Dynamics of Transient Neuronal Signal in Mammals Cold Receptor

**DOI:** 10.1155/2016/2754249

**Published:** 2016-09-28

**Authors:** Firman Ahmad Kirana, Husin Alatas, Irzaman Sulaiman Husein

**Affiliations:** ^1^Theoretical Physics Division, Department of Physics, Bogor Agricultural University, Kampus IPB Darmaga, Jl. Meranti, Bogor 16680, Indonesia; ^2^Research Cluster for Dynamics and Modeling of Complex Systems, Faculty of Mathematics and Natural Sciences, Bogor Agricultural University, Kampus IPB Darmaga, Jl. Meranti, Bogor 16680, Indonesia; ^3^Applied Physics Division, Department of Physics, Bogor Agricultural University, Kampus IPB Darmaga, Jl. Meranti, Bogor 16680, Indonesia

## Abstract

We propose a theoretical model consisting of coupled differential equation of membrane potential phase and temperature for describing the neuronal signal in mammals cold receptor. Based on the results from previous work by Roper et al., we modified a nonstochastic phase model for cold receptor neuronal signaling dynamics in mammals. We introduce a new set of temperature adjusted functional parameters which allow saturation characteristic at high and low steady temperatures. The modified model also accommodates the transient neuronal signaling process from high to low temperature by introducing a nonlinear differential equation for the “effective temperature” changes which is coupled to the phase differential equation. This simple model can be considered as a candidate for describing qualitatively the physical mechanism of the corresponding transient process.

## 1. Introduction

Mammals complex thermoreceptor systems consisting of free nerve ending fibers are located in the dermis, muscle, skeleton, liver, and hypothalamus [1]. It is a phasic receptor which is active when there is a change in environmental temperature and rapidly becomes steady when reaching the stable temperature. Based on its characteristics with respect to the temperature level, it can be classified into warm or cold receptor [2, 3], which is, respectively, sensitive to high or low temperature relative to the normal body temperature, characterized by its way in delivering the neuronal signals. The corresponding neuronal signals are delivered in the form of bursting, that is, rhythmic of action potential consisting of spikes and punctuated by periods of inactivity [4, 5]. Their characteristics depend strongly on the associated temperature levels.

In this report, we focus our discussion on the dynamics of mammals cold receptor. In a low temperature condition, the corresponding neuronal signals produce periodic bursts with uniform duration and slow oscillation characteristic, but with nonuniform spike frequencies for each burst. When the temperature is raised up by a quasistatic process, the amount of spikes per burst tends to decrease forming a periodic single spike or beating. At a relatively higher temperature, the spike pattern becomes aperiodic; namely, it can also exhibit either double spike or stochastically phase-locked spike (skipping) phenomenon [3]. An experimental study on the static and dynamic discharge of a specific mammals cold receptor, that is, cat's lingual nerve, has been comprehensively conducted by Braun et al. [4]. In particular, they showed that the dynamical response of the associated cold receptor is different for various temperature transitions between 10°C and 40°C.

Nowadays, many models have been proposed to explain the dynamical characteristics of mammals cold receptor. One of the most profound models is the conductance-based model which relies on the conductance voltage-dependent phenomenon due to the existence of Na^+^ and K^−^ ions. For example, Braun et al. [6] in their report had discussed a Hodgkin-Huxley voltage-conductance type equation in their attempt to understand the role of nonlinearity and noise on the dynamics of nerve cell membrane through mammals cold receptor data. Another conductance-based cold receptor model was also discussed in different reports [7, 8].

In the meantime, there is a certain type of ion channel called transient receptor potential melastatin 8 (TRPM8) that plays an important role in delivering the cold receptor neuronal signal (see [9] for review). The role of TRPM8 has been shown experimentally in thermosensation mechanism in mice as discussed in [10–12]. Very recently, a conductance-based model which includes the role of TRPM8 ion channel has been proposed by Olivares et al. and showed a good agreement with the experimental data found from the cold receptor of mice [13]. The corresponding Olivares model successfully resembled the experimental data of increasing the firing rate for quasistatically increasing exposed temperature protocol.

Apart from those conductance-based models, a fully ionic model has been proposed by Longtin and Hinzer [5], which discussed the stochastic action potential phase model specifically for a cat's lingual cold receptor. This model was further simplified by Roper et al. [14], namely, by introducing a simplified phase differential equation. It was demonstrated that the corresponding model was able to approximate the Longtin-Hinzer model for temperature interval 17.8°C to 40°C. Compared to the conductance-based model, Roper's model [14] offered a relatively simple mathematical description. However, we discovered that this model did not lead to a realistic description on phenomena that occurred in higher or lower temperature conditions.

Based on this fact, in the present report we discuss a possible modification on the corresponding Roper's model for the nonstochastic limit by introducing a new functional form of parameters that appeared in the corresponding model. Furthermore, we also discuss an extension of the corresponding modified model to accommodate the dynamical response of neuronal signals during a transition process from high to low temperature condition. This dynamical model is able to explain the phenomenon of sudden increasing amount of spikes per burst due to decreasing temperature, which is followed by a gradual decreasing of the corresponding amount of spikes per burst until the receptor reaches a steady condition at the lower temperature [4, 15]. We explain this phenomenon by considering an additional differential equation to describe the temperature dynamics, which is coupled to the associated phase differential equation.

We organize the report as follows: Section 2 discusses the phase model for the case of the steady temperature condition. The modified models for static temperature and dynamic transient process from high to low temperature are given in Section 3, namely, by defining a new set of functional parameters in the corresponding phase differential equation and introducing a new differential equation of temperature coupled to the phase differential equation and we focus our discussion on the characteristics of spike per burst, burst period, and interspike interval. We end this report with a conclusion in Section 4. Comprehensive discussions regarding the biological and chemical related properties of the corresponding cold receptor have been given in detail previously [4, 5, 14], such that in this report we only focus on the modified mathematical model.

## 2. Model and Method

The corresponding nonstochastic phase differential equation for steady condition of neuronal signaling at a specific temperature developed previously by Roper et al. [14] is given as follows:

(1) $$\begin{array}{c} \frac{d\theta }{dt}=F\left(\;t,\theta \;\right) \end{array}$$

with

(2) $$\begin{array}{c} F\left(\;t,\theta \;\right)=f_{1}\left(\;t\;\right)+f_{2}\left(\;t\;\right)\cos\theta , \end{array}$$

where

(3) f1t=b−Acos⁡Ωt,

(4) f2t=1+Acos⁡Ωt.

Here, the symbol *θ* represents the phase of membrane potential in the trigger region, in which its full rotation describes the generation of an action potential [14]. The parameter *b* is related to the modulation of the mean potential of the cell, while the term *A*cos⁡(*Ωt*) is a zero mean periodic term that oscillates with the frequency *Ω*, with *A* as the corresponding magnitude. The function *F*(*t*, *θ*), with an inverse time unit, describes the dynamics of the corresponding neuronal signal bursting [14].

**Figure 1:**
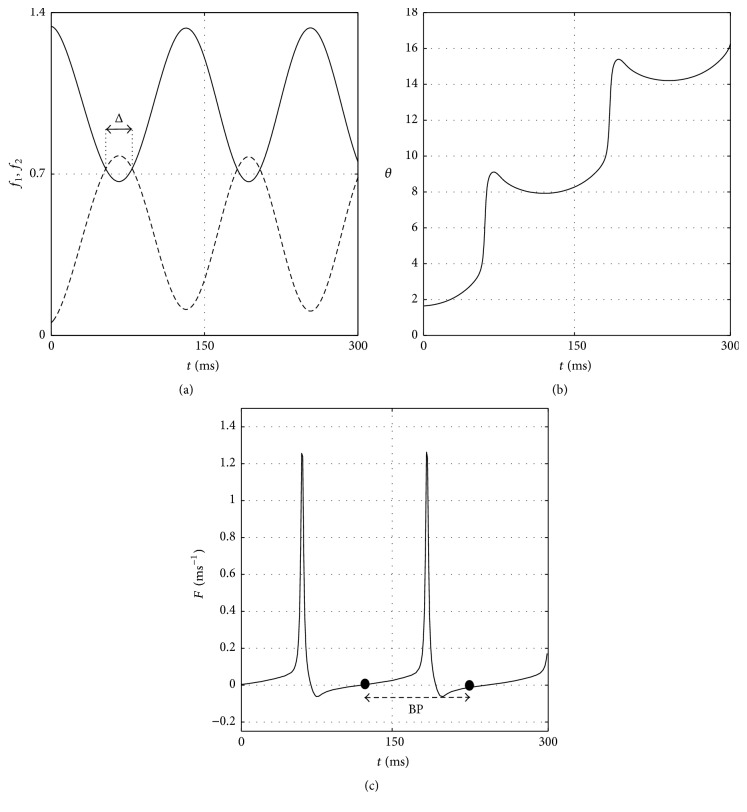
(a) The plot of *f*_1_ (solid curve) and *f*_2_ (dash curve) functions with Δ denotes the maximum width of overlap area associated with SB along with (b) phase of membrane potential and (c) the corresponding neuronal signal. BP denotes the burst period.

It is seen that there are two important terms in (2), namely, *f*_1_ and *f*_2_ functions, as given by (3) and (4), respectively. The burst occurs when *f*_1_ > *f*_2_, where the average amount of spikes in each burst is proportional to the maximum width of the overlap area of both curves, denoted by Δ, as exemplified in Figure 1(a) along with the corresponding phase of membrane potential (*θ*), which is found by solving (1), and neuronal signal bursting (*F*) functions as shown in Figures 1(b) and 1(c), respectively. It is obvious that the amount of spikes per burst can be controlled by changing the value of *b* and *A* as well as *Ω*  which also determine the period between two consecutive bursts. It was assumed previously [14] that these parameters are of linear functional forms of temperature as follows:

(5) b=b0−b1T,

(6) A=A0+A1T,

(7) Ω=Ω0+Ω1T

with *b*_0_, *b*_1_, *A*_0_, *A*_1_, *Ω*_0_, and *Ω*_1_ being constants to be determined. This assumption was aimed at yielding a decreasing period between two consecutive bursts when the temperature increases through a quasistatic process. In their work, Roper et al. defined the value of each parameter as follows: *b*_0_ = 0.675 ms^−1^, *b*_1_ = 0.007 ms^−1^, *A*_0_ = 0.3 ms^−1^, *A*_1_ = 0.001 ms^−1^, *Ω*_0_ = −*π*/150 ms^−1^, and *Ω*_1_ = *π*/1500 ms^−1^. We used all these parameters at *T* = 35°C to depict the example shown in Figure 1.

Based on the above formulation, it is clearly seen that these linear assumptions will lead to an unrealistic scenario at the high and low temperature conditions, since all those parameters are not saturated at these limits. Therefore, it is reasonable to assume that phenomenologically at those temperatures the neuronal signals become saturated since in that range the receptor becomes less sensitive [16]. It is interesting to note that this model can also be further developed to describe the transient transition from high to low temperatures as previously reported by Ring and de Dear [15]. For this, we propose assuming that the corresponding temperature should be considered as a function of time with Morse-like characteristic described by a differential equation which is coupled to the corresponding phase differential equation given by (5). To study the dynamical characteristics of this model, we numerically solve the related coupled differential equations by means of standard Runge-Kutta method.

## 3. Results and Discussion

### 3.1. Modified Phase Model for Steady Temperature Condition

To develop a more realistic model, we consider the modification of *b*, *A*, and *Ω* parameters by introducing the following tanh functional forms:

(8) b=b0−b1tanh⁡CTeff−T−,A=A0+A1tanh⁡CTeff−T−,Ω=Ω0+Ω1tanh⁡CTeff−T−

which exhibit sigmoidal saturation characteristic at relatively high and low temperatures. Here, *C* and $\overset{-}{T}$ are parameters to be adjusted. We denote the parameter *T*_eff_ as an “effective temperature” for describing the dynamics of neuronal signal during the transient process from high to low environmental temperature. The meaning of this parameter will become clear in later discussion (see Section 3). Obviously, the functional parameter forms given by (8) will lead to saturated behavior of neuronal signal at high and low temperatures. By considering the same values with that used previously [14] for a temperature interval of 40°C to 15°C and *Ω* → 0 at *T*_eff_ → −∞ we found *C* = 0.055/°C and $\overset{-}{T}$ = 33.75°C, while the other parameters are set to *b*_0_ = 0.4475 ms^−1^, *b*_1_ = 0.1575 ms^−1^, *A*_0_ = 0.3325 ms^−1^, *A*_1_ = 0.0225 ms^−1^, *Ω*_0_ = 3*π*/200 ms^−1^, and *Ω*_1_ = 3*π*/200 ms^−1^.

**Figure 2:**
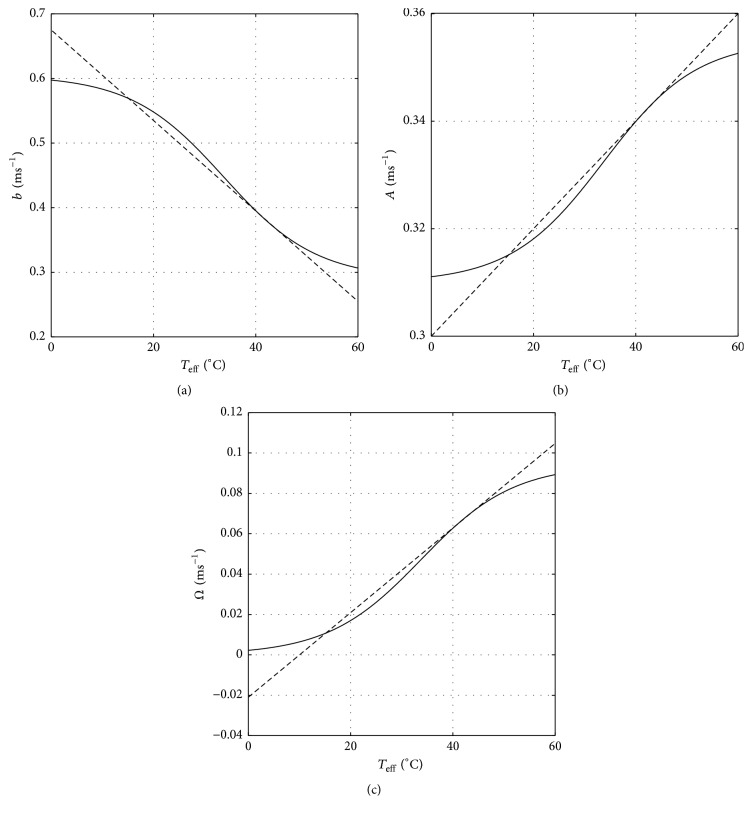
Comparison between the previous set of functional parameter forms (dash curve) with the new one (solid curve) for (a) *b*, (b) *A*, and (c) *Ω* parameters.

Demonstrated in Figure 2 is the comparison between the previous set of functional parameter forms and the new ones. It should be realized that, for the actual cases, those functional forms should be adjusted using experimental data of the associated spiking and bursting neuronal signaling phenomena in low and high temperature conditions. Indeed, it is also important to note that one can choose different type of functional forms. A nonsigmoidal functional form was previously proposed in [17] which was aimed at mimicing the bifurcation characteristics of the conductance-based Huber-Braun model [7].

**Figure 3:**
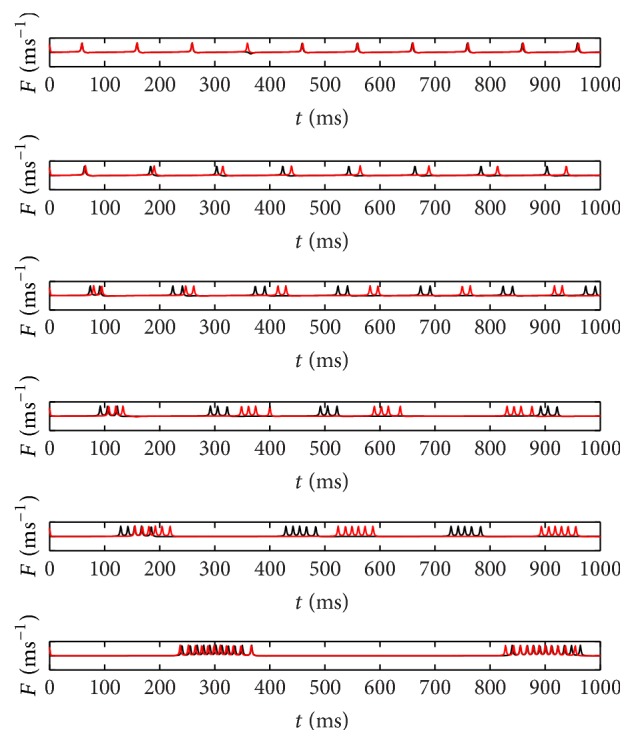
Bursting characteristics resulted from previous model (red curve) [14] and present modified model (black curve) for, from top to bottom panels, *T*_eff_ = 40°C, 35°C, 30°C, 25°C, 20°C, and 15°C.

**Figure 4:**
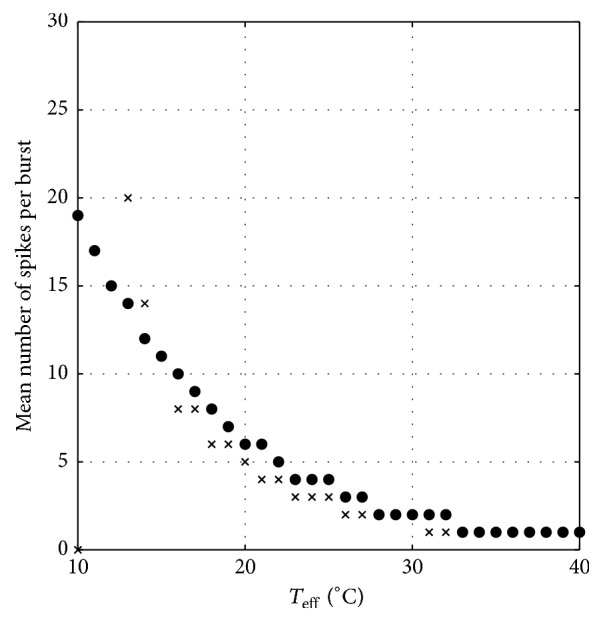
Mean SB at steady condition resulted from previous model (cross) [14] and present modified model (solid circle).

The corresponding neuronal signals at steady temperatures for *T*_eff_ = 40°C to 15°C, which are compared to the previous reported results [14], are depicted in Figure 3. Here the function *F* of (2) is calculated by solving first *θ* function in (1) and then the solution is inserted into the corresponding equation. The average amount of spikes per burst (SB) for both functional forms is given in Figure 4. It is observed that, at low temperature condition, the tanh functional forms exhibit a more reasonable amount of spike per burst than the linear functional forms of Roper's model [14] that exhibits higher amount as demonstrated in Figure 4. Indeed, there are some discrepancies between both linear and tanh functional forms since both assumptions do not perfectly coincide as clearly shown in Figure 2. But indeed, both forms share qualitatively similar bursting and spiking characteristics. As shown by the figures, it is important to realize that, for decreasing *T*_eff_, the amount of spikes per burst is increasing.

**Figure 5:**
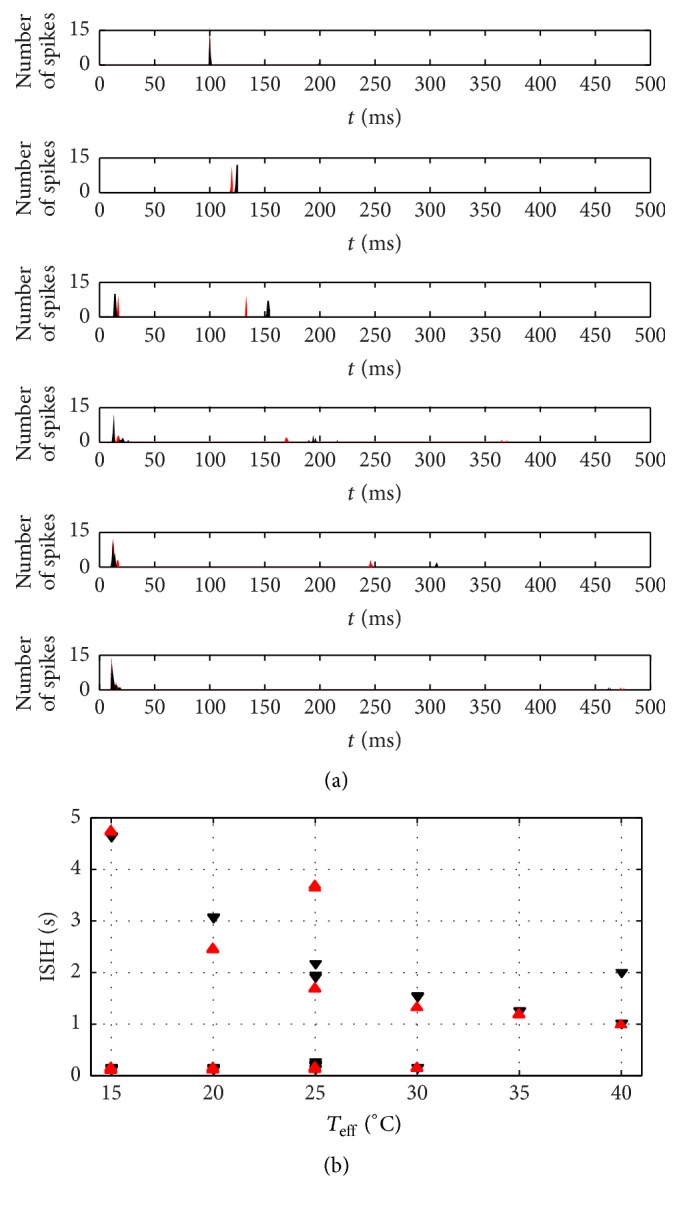
(a) From top to bottom panel, interspike interval histogram (ISIH) of the Roper (red curves) and modified (black curves) model at steady conditions for *T*_eff_ = 40°C, 35°C, 30°C, 25°C, 20°C, and 15°C. (b) Multivalued characteristics of ISIH for Roper (red arrow-head) and modified (black arrow-head) model as a function of *T*_eff_.

The other important characteristic, namely, the interspike interval histogram (ISIH) of the neuronal signal at the corresponding different temperatures for the tanh functional forms, is given in Figure 5(a) for both Roper and the present modified model, along with its plot as a multivalued function of *T*_eff_ in Figure 5(b). The ISIH shows the existence of beating and skipping at high temperature condition which are indicated by the presence of large intervals. Clearly, the modified model exhibits a bit different characteristics than the original Roper's model.

### 3.2. Model for Transient Transition Process

**Figure 6:**
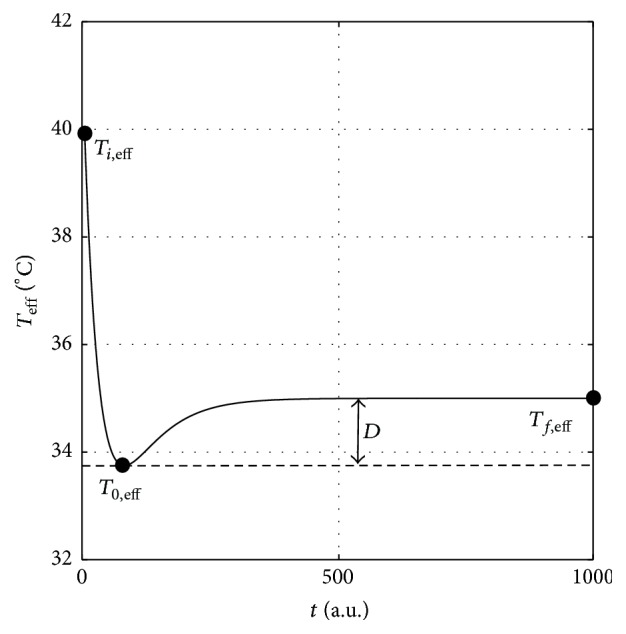
Illustration of a Morse-like function.

During a transient transition from high to low temperature, the existence of a peak response with relatively large amount of spikes per burst at a certain time was shown experimentally as the transition process begins as reported in [15, 18]. Based on the burst characteristic at steady temperatures as exemplified by Figure 3, we suspect that this condition might be perceived by the brain to occur at a temperature lower than the final temperature. In the meantime, another experimental result demonstrated that the period between two consecutive bursts during the transient transition process is higher than the period at final temperature [4]. To model the corresponding dynamical response, we consider first a conjecture that the effective temperature *T*_eff_ in our formulation is a function of time in the following Morse-like function [19]:

(9) $$\begin{array}{c} T_{\text{eff}}=D{\left\{\;1-\exp\left[\;-a\left(\;t-\overset{-}{t}\;\right)\;\right]\;\right\}}^{2}+T_{0,\text{eff}}, \end{array}$$

where *T*_eff_ and *T*_0,eff_ are effective temperature at time *t* and its lowest value, respectively, whereas $\overset{-}{t}$ denotes the time when *T*_eff_ = *T*_0,eff_. Graphically, the parameter *D* determines the depth of Morse-like curve as illustrated in Figure 6, while *a* denotes its effective width. The corresponding Morse-like function is chosen because it is a mathematically well-defined function with no singularity. Phenomenologically, it is likely to be the best geometrical shape to describe the corresponding transient response characteristics among other similar functional forms such as the Lennard-Jones [20], the Buckingham Exponential-6 [21], and the Mie potential functions [22]. All these functions are commonly used to describe the molecular interactions [22]. In contrast to the Morse function, the other three functions contain a singularity.

It should be emphasized that the existence of the abovementioned peak response with large amount of spike per burst during the transient transition is the reason to define the term “effective temperature” as a tuning factor in our formulation based on the following argument: as shown in Figure 3, the amount of SB for low temperature is larger than the higher one. At the same time, a sudden increasing amount of SB occurs due to decreasing temperature, which is followed by a gradual decrease of SB until the receptor reaches a steady condition at the lower temperature [4, 15]. From all these facts, we therefore propose that the *T*_eff_ functional parameter, with its curve given in Figure 6, should be considered as a dynamical tuning factor that is needed to describe the dynamics of the related neuronal signal propagation.

It is easy to prove that the function given by (9) satisfies the following differential equation:

(10) $$\begin{array}{c} \frac{dw}{dt}=a\left(\;D^{1/2}-w\;\right), \end{array}$$

where

(11) $$\begin{array}{c} w^{2}=T_{\text{eff}}-T_{0,\text{eff}}. \end{array}$$

In the ensuing discussion, we choose to solve (10) numerically rather than using (9) in order to explain the corresponding peak response phenomenon. It should be noted that, to ensure the corresponding numerical solution of (10) is the Morse-like function as given by (9), one should consider a negative initial condition for *w*; that is, $w\left(0\right)=-\sqrt{T_{\text{eff}}\left(0\right)-T_{0,\text{eff}}}$.

We expect the parameters *a* and *D* can be determined experimentally. However, in our calculation, we assume that the parameter *D* is fixed to

(12) $$\begin{array}{c} D=\frac{-\left(T_{f,\text{eff}}-T_{i,\text{eff}}\right)}{4}, \end{array}$$

where *T*_*f*,eff_ and *T*_*i*,eff_ denote the final and initial effective temperature, respectively, such that *T*_0,eff_ in (9) and (11) is fixed to

(13) $$\begin{array}{c} T_{0,\text{eff}}=\frac{\left(5T_{f,\text{eff}}-T_{i,\text{eff}}\right)}{4}. \end{array}$$

**Figure 7:**
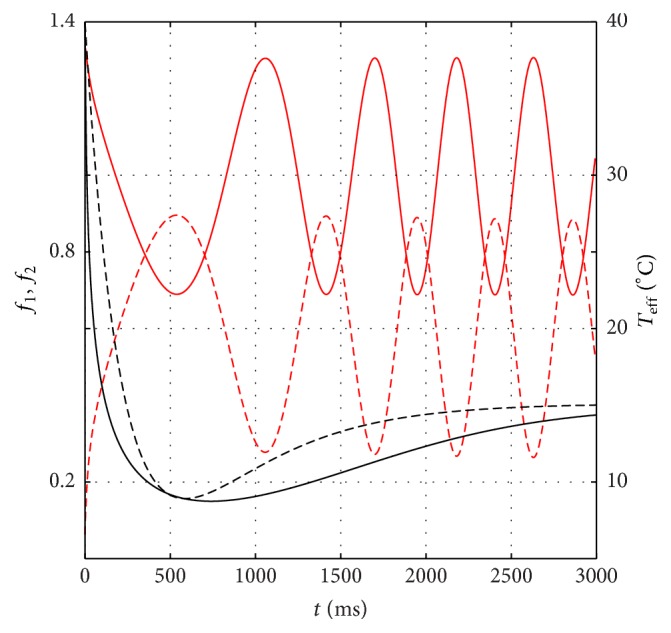
Matching condition between the first bursting and lowest effective temperature from transient transition from *T*_*i*,eff_ = 40°C to *T*_*f*,eff_ = 15°C. The Morse-like function found by solving (9) (dash-black curve) and (14) (solid-black curve).

**Figure 8:**
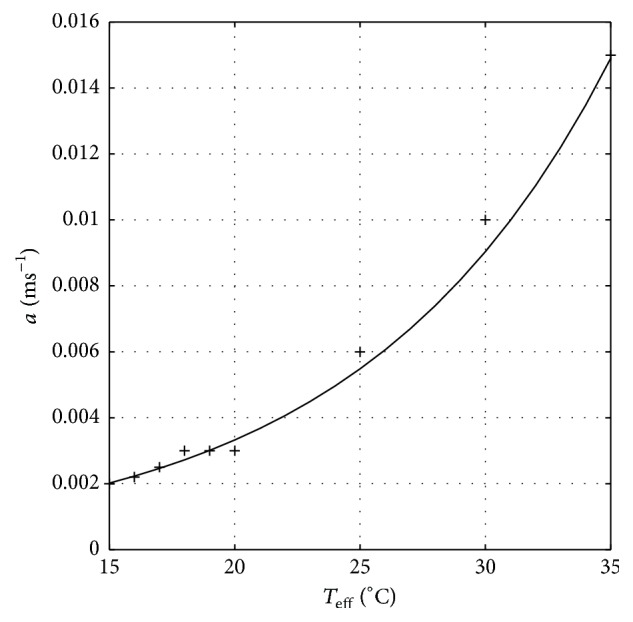
Approximate function of *a*(*w*) (solid curve) based on values of *a* (solid diamond) found by solving (9) from several transient transition processes.

Indeed, one can assume different values for this parameter and it is clear that different *T*_0,eff_ leads to a different $\overset{-}{t}$ in (9). On the other hand, it is reasonable to assume that the parameter *a* in (10) should be expressed as a function of *w*, that is, *a* ≡ *a*(*w*), because it is natural to think that the shape of the associated Morse-like function is different in different transition process. To formulate the corresponding expression, first we plot functions *f*_1_ and *f*_2_ given by (3) and (4) and adjust the value of *a* in (10) to meet a matching condition, which is indicated by the coincidence between the first overlaps width of both functions (denoted by Δ in Figure 1) and the lowest effective temperature *T*_0,eff_. Exemplified in Figure 7 is the associated matching condition for the dynamical response from *T*_*i*,eff_ = 40°C to *T*_*f*,eff_ = 15°C. For this transition, we found that the matching condition occurs at *a* = 0.002 ms^−1^. The calculation result for this parameter from *T*_*i*,eff_ = 40°C to various *T*_*f*,eff_ is given in Figure 8. Using a standard fitting procedure, it is found that all those values can be approximated by the following function:

(14) $$\begin{array}{c} a\left(\;w\;\right)=a_{0}exp{\left[\;\alpha \left(\;w^{2}+T_{0,\text{eff}}\;\right)\;\right]}^{} \end{array}$$

with *a*_0_ = 4.5 × 10^−4^ ms^−1^ and *α* = 0.1/°C, while *T*_0,eff_ is given by (12). Therefore, it is clear that the differential equation (10) should be rewritten as follows:

(15) $$\begin{array}{c} \frac{dw}{dt}=a_{0}\exp\left[\;\alpha \left(\;w^{2}+T_{0,\text{eff}}\;\right)\;\right]\left(\;D^{1/2}-w\;\right) \end{array}$$

which is coupled to (1) through parameters given by (8). It is important to note that the *D* parameter and *a*(*w*) function phenomenologically correspond to the characteristics of the burst period and amount of SB around the matching condition, respectively. Therefore, as mentioned previously, it is obvious that these two parameters should be determined experimentally by observing the spiking and bursting characteristics similar to what was done in [4].

**Figure 9:**
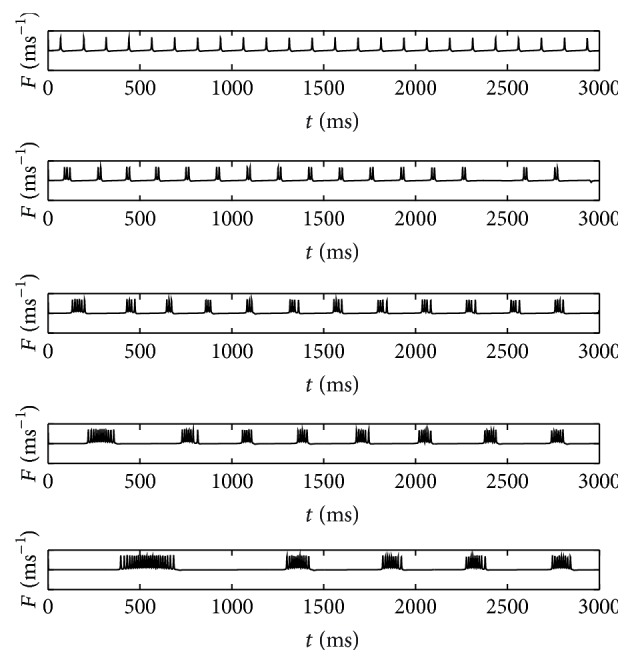
Bursting characteristics resulting from modified model for a transient transition from (top to bottom panels) *T*_*i*,eff_ = 40°C to *T*_*f*,eff_ = 35°C, 30°C, 25°C, 20°C, and 15°C.

**Figure 10:**
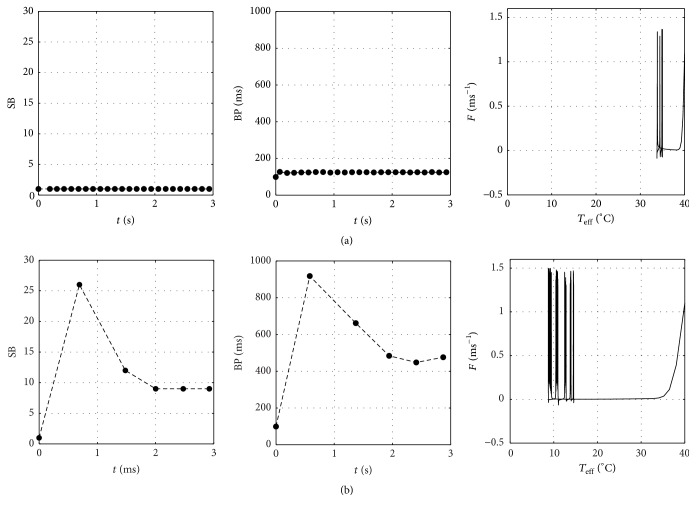
Amount of SB (left panel) and BP (mid panel) resulting from present modified model (left panel) and parametric plot in phase-plane of *T*_eff_ and *F* functions (right panel) for transition from *T*_*i*,eff_ = 40°C to (a) *T*_*f*,eff_ = 35°C and (b) *T*_*f*,eff_ = 15°C. Each SB and BP are plotted at the end-time of a peak of burst last spike and at the midtime of two points as shown in Figure 1(c), respectively.

Using this new model, the simulation results of transition processes from *T*_*i*,eff_ = 40°C to *T*_*f*,eff_ = 35°C, 30°C, 25°C, 20°C, and 15°C are depicted in Figure 9. Given in Figure 10 are the SB for transition to *T*_*f*,eff_ = 35°C and 15°C, along with the corresponding burst period (BP) as defined in Figure 1(c), which is qualitatively similar to that given in [4]. The parametric plot in phase-plane of *T*_eff_ and *F*(*t*, *θ*) to give a more clear description is also given in Figure 10. It is shown that the SB and BP characteristics exhibit pronounced transient feature for transition to *T*_*f*,eff_ = 15°C. In the meantime, the occurrence of dense patterns of bursting process when *T*_eff_ < *T*_*f*,eff_ is demonstrated, justifying the existence of previously discussed peak response phenomenon as shown experimentally in [4] during a transient time. In contrast, it is interesting to note that a monotonous response characteristic is exhibited during *T*_eff_ > *T*_*f*,eff_.

An example of the approximate Morse-like function found from (15) is given in Figure 7, which shows the same position of the related matching condition compared to Morse-like function found by solving (10) numerically. It is clearly shown that, right after the transition process begins, namely, at the first burst, the amount of the corresponding spikes is larger than the next bursts. As a consequence of choosing the saturated tanh functional forms in the model parameters as given by (8), we found that the change of the SB at matching condition is at a reasonable level, especially in the case of *T*_*f*,eff_ = 15°C, where *T*_0,eff_ < 15°C.

**Figure 11:**
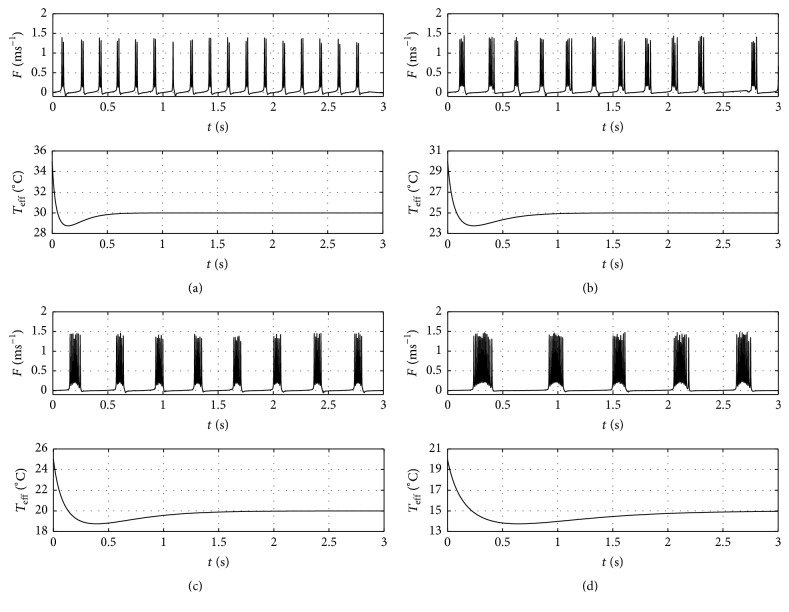
The burst phenomenon (top panel) and *T*_eff_ function (bottom panel) for transition from (a) *T*_eff_ = 35°C to 30°C, (b) *T*_eff_ = 30°C to 25°C, (c) *T*_eff_ = 25°C to 20°C, and (d) *T*_eff_ = 20°C to 15°C.

**Figure 12:**
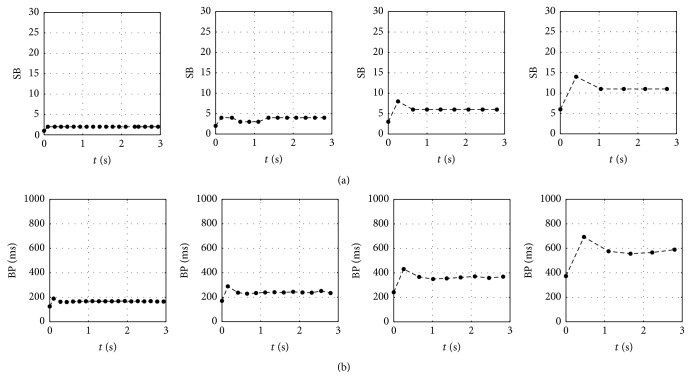
(a) SB and (b) BP resulted from present modified model (solid circle) for transition (left to right panel) from *T*_eff_ = 35°C to 30°C, *T*_eff_ = 30°C to 25°C, *T*_eff_ = 25°C to 20°C, and *T*_eff_ = 20°C to 15°C. Each SB and BP are plotted at the end-time of a peak of burst last spike and at the midtime of two points as shown in Figure 1(c), respectively.

To validate this modified model with experimental data, we focus on comparing qualitatively the SB and BP characteristics with the results reported by Braun et al. [4]. Given in Figure 11 are the spiking and bursting phenomena for the transition similar to what was discussed in [4] along with the associated *T*_*f*,eff_ function. We calculate the SB of four different *T*_*f*,eff_ transition intervals, as well as the BP parameter. The results are shown in Figure 12. It is interesting to note that qualitatively the corresponding SB characteristic exhibits fairly similar trend with the experimentally found SB figure in Figure 5 in [4], while it is seen that graphically that BP exhibits similar characteristics with SB. Note that the transient characteristics are demonstrated significantly in the cases of low temperature transitions, that is, *T*_*i*,eff_ = 25°C to *T*_*f*,eff_ = 20°C and *T*_*i*,eff_ = 20°C to *T*_*f*,eff_ = 15°C. This feature can be explained as a consequence of *T*_eff_ function with wider profile due to larger *a* value as shown in Figure 11.

On the other hand, in comparison with Olivares's model [13], taking into account the role of TRPM8 ion channel transient current to explain the phenomenon of increasing firing rate (which indicates the increasing of SB) as exposed temperature decreases, this modified model offers a different perspective to the transition mechanism from high to low temperature, namely, by introducing the “effective temperature” (*T*_eff_) functional parameter as a dynamic tuning factor that coupled to the phase of membrane potential. As discussed previously, although the physical meaning of this dynamical parameter is not clearly understood at this moment, we proposed that this parameter might be interpreted as an actual temperature being perceived by the mammal brain and it is likely reasonable to assume that the corresponding transient *T*_eff_ function is related to the complex role of TRPM8 ion transient channel. Indeed, this hypothesis should be separately investigated.

Furthermore, although our model is able to describe the existence of peak response at matching condition, however, it should be noted that the model leads to the increasing pause duration or the time distance between two consecutive bursts at the corresponding condition, while in reality this is not the case as reported previously [4]. This problem is a bit complicated to be solved and we suggest it can be overcome by defining new functional forms for parameters given by (8). The other problem with our model is related to the value of *D* in (11) where in our calculation it was considered to be fixed. We expect this parameter can be determined experimentally, and this is beyond the scope of our study.

To this end, apart from the above mentioned problems, it is also realized that this modified model should be improved further, since the related effective temperature differential equation given by (15) does not take into account the influence of phase of membrane potential. We suggest that fully coupled differential equations that accommodate this feature will likely be able to give a good quantitative explanation of the dynamics and characteristics of neuronal signals of the corresponding cold receptors. This issue could be a challenging topic for future investigation.

## 4. Conclusion

We have discussed a modified Roper's model for describing the characteristics of neuronal signaling in mammals cold receptor, especially for the temperature transition processes. The model consists of coupled phase-temperature nonlinear differential equations equipped with a set of functional parameters that saturate at low and high temperature. It was shown that our modified model is able to describe the experimental fact that the characteristics of neuronal signal in a transient transition process from high to low temperature exhibit the existence of large amounts of spikes per burst right after the process initiated, namely, by introducing the new functional parameter “effective temperature,” which plays a role as a dynamical tuning factor to explain the corresponding phenomenon. We propose that this dynamical tuning factor might be interpreted as a perceived temperature by the mammal brain in which its perception of temperature at $\overset{-}{t}$ has the lowest value, while *T*_*i*,eff_ and *T*_*f*,eff_ are coincides with the environmental temperatures. Certainly, it is intriguing to further examine experimentally whether this interpretation is correct or not. For instance, by observing the related mammal brain activity that corresponds to the temperature perception. Further studies should be conducted in order to overcome a few problems that still exist. However, this modified model can be considered as a dynamic simple alternative candidate to complex ionic models to describe qualitatively the transient transition from high to low temperature of the mammals cold receptor.
